# Editorial: Heterologous production of high value metabolites in plants and microbes

**DOI:** 10.3389/fpls.2023.1223033

**Published:** 2023-06-14

**Authors:** Vishwas Anant Bapat, Polavarapu. B. Kavi Kishor, Suprasanna Penna

**Affiliations:** ^1^ Department of Biotechnology, Shivaji University, Kolhapur, India; ^2^ Department of Genetics & Biotechnology, Osmania University, Hyderabad, India; ^3^ Amity Centre for Nuclear Biotechnology, Amity Institute of Biotechnology, Amity University of Maharashtra, Mumbai, India

**Keywords:** plants, microbial systems, secondary metabolites, bioengineering, plant natural products, transgenic plants

Plants and microbial derived secondary metabolites have multitude of functions in growth, development and resilience to environmental stresses. These secondary metabolites are also exploited for medicinal, cosmeceutical and nutraceutical applications. Based on the demand and the economic feasibility, strategies for large-scale production have been designed using microbial and plant-based production platforms. Methods of metabolic engineering and synthetic biology have now become useful for engineering metabolic and biosynthetic pathways in plant and microbial systems as eco-friendly alternative approaches (Pham et al., 2019; Schillberg et al., 2019; Bapat et al., 2022). Plant cell cultures have become the candidates of choice for the production of bioactive compounds as they perform posttranslational modifications and can be manipulated using genomic techniques (Bapat et al., 2023). Bioactive metabolites used in healthcare and in other industrial processes have been derived from different biological sources. Among an array of the natural products, the main thrust has been to obtain therapeutic proteins and other useful molecules from the natural resources. The market size of global microbial products is expected to be worth around USD 302 billion by 2030, whereas, plant-derived drug market is projected to grow at a rate of more than 6.1% over the period 2019-2026. More traditional**/**natural drug manufacturers are gearing up with a wide spectrum of products that are effective as therapeutic molecules. Hence, a phenomenal rise for the plant-derived pharmaceuticals can be expected during the next decade. Remarkable achievements have been made to enhance the production of pharmaceutically important compounds using biotechnological methods. The recent advancement in the application of *in vitro* techniques for the production of cosmetics is a good example contributing to the cosmeceutical industry (Gomes et al., 2020; Morikawa et al., 2020).

In view of the tremendous potentials of natural products, present collection of articles is focused on strategies of heterologous synthesis of high value metabolites. High value metabolites of the natural origin have become now inevitable for a wide range of applications in human health and industry. Several potential biotechnological and bioengineering platforms for the making of industrially relevant biochemicals, pharmaceuticals, and biofuels, have been employed for the products, which are often not easy to produce by the conventional technologies. Successful heterologous synthesis of metabolites depends on the availability of highly efficient promoters for heterologous expression, codon optimization, suitable selection of markers, co-transformation efficiency, and multiple expression constructs (Ghag et al., 2021). Consequent to the availability of genome sequences, and advances in systems and synthetic biology, new options have emerged for designing metabolic engineering strategies for the synthesis of metabolites on a commercial scale. This collection includes articles based on the latest developments in the field of heterologous synthesis of plant metabolites. Each article is structured around a comprehensive conceptual framework that integrates a broad range of regulatory factors that define and contextualize the understanding of the future work.

## Production of high value metabolites in plants

Badim et al., showed that a grapevine transcription factor *VviNAC17* activated by abscisic acid has been shown to modulate the secondary metabolism in grape berry skins. The authors have demonstrated that the constitutive expression of a transcription factor significantly induced the synthesis of flavonoids and other phenolics in transgenic grape berry cells through upregulation of several genes of the phenylpropanoid (*VviPAL1*), stilbenoid (*VviSTS1*) and flavonoid pathways (*VviDFR, VviLAR1, VviANR, VviLDOX, and VviUFGT1*), as well as anthocyanin vacuolar transport and accumulation (*VviGST4 and VvMATE1*) in the *VviNAC17*-overexpressing transgenic cells. Regulation of the metabolic pathways is crucial to ensure better berry quality, which consequently is translated into better wine quality and production of better products by the winemaking industry. Authors have opined that *VviNAC17* and the Gamay Fréaux grape berry cells can be used to overproduce the secondary metabolites of benefit to agrifood, nutraceuticals and even the cosmetics industry.

Yao et al. designed the biosynthetic modules for efficient production of high value plant natural products, genistein and scutellarin, based on the transient expression system of *Nicotiana benthamiana* and using synthetic biology strategies. The production of scutellarin in a heterologous plant by Yao et al. is a first report. The work has highlighted that the sustainable production of genistein and scutellarin in tobacco plants offers a novel strategy for the industrial scale production of high value plant natural products. Several plant natural products possess multitude of bioactive properties of significance to healthcare. Industrial-scale plant factories are now in application stage for commercial manufacturing of vaccines or natural products (Lee et al., 2023). For example, the *Medicago* plant factory has the capability to yield 10 million doses of pandemic H5N1 vaccine per month (Lomonossoff and D’Aoust, 2016).

Hou et al. established a production system for fibroblast growth factor FGF21 in both transgenic seeds and leaves of commercial *N. tabacum* cultivars (Virginia Golta and SL632 known for high leaf and seed biomass). The bioavailability and bioactivity through oral delivery in a first *in vivo* trial in mice indicated that FGF21 can be used for treating non-alcoholic steatohepatitis disease. This study highlighted the possible medication of non-alcoholic steatohepatitis by oral administration of nTf338-FGF21-PLUS containing plants. Non-alcoholic steatohepatitis is a widespread disease with no therapeutic interventions. The fibroblast growth factor FGF21 can reverse this liver dysfunction and a directed transport to liver via oral delivery portal vein can be a better approach than systemic administration which often results in intestinal absorption.

Dietary lignans derived from plant based resources have diverse health benefits. Koyama et al., reviewed the work on the heterologous production of beneficial lignans using different transgenic plant systems. The production of (+)-sesamin was achieved using sesame CYP81Q1 gene in *Forsythia* plants, and co-expression of *Podophyllum* (-)-podophyllotoxin-biosynthetic enzyme genes *CYP719A23, OMT3, CYP71CU1, OMT1, and 2-ODD* resulted in podophyllotoxin in *Nicotiana benthamiana*. Authors have also highlighted the significance of introduction of novel genes, elicitation of precursor accumulation and optimizing growth conditions of heterologous plants for the production of beneficial lignans. This study on the production of beneficial lignans in cultured cells and hairy root lines of heterologous host plants can be a good option since production of lignins like (+)-sesamin and (-)-podophyllotoxin in plants is often constrained due to environmental stresses.

Park et al., studied the action of MEP and MVA pathway genes, single or combined, for increasing linalool and costunolide production in *Nicotiana benthamiana.* Transgenic *N. benthamiana* plants with all the selected genes were transiently overexpressed thereby increasing linalool and costunolide production. The work highlighted, the transient expression of pathway genes which enhanced, linalool and costunolide production in the transgenics. Further, the study necessitates the approach of synthetic metabolic engineering through the identification of crucial genes for synthesis, regulatory control for the biosynthetic conduit of desired metabolites, need for a plant system with transformation efficiency and, ability for biomass accumulation for industrial scale up of linalool and costunolide in heterologous plant systems. The monoterpenoid linalool and sesquiterpenoid costunolide have applications as essential oils, feedstocks, food additives and pharmaceuticals.

Kulshreshtha et al., reviewed the progress made in plant-based expression platforms to produce high value metabolites and proteins. They discussed developments in the area of recombinant technology especially CRISPR/Cas9 system, plant cell, tissue, and organ culture, and pointed out the remarkable progress that has been made to increase the expression of recombinant proteins and important metabolites in the plants. Additionally, approaches like stabilization of RNA transcripts, optimization of protein translation, engineering of proteins for their constancy, and targeting of proteins to subcellular locations have been described in light of their relevance and potentials for heterologous production of high value metabolites using plant expression systems.

Significant progress in the design and validation of heterologous plant and microbial platforms has generated great interest on the production and scale up of some of the bioactive metabolites which are poised to enter into commercialization. For many of the bioactive metabolites, there is a need to unravel the complete biosynthetic pathways and the associated genetic machinery including the spatial and temporal regulatory hubs. This Research Topic will help researchers to produce high value compounds of pharmaceutical importance on a commercial scale. Breakthroughs in the synthetic biology, can lead to the synthesis of pharmaceutically important plant metabolites in a tailored fashion, in heterologous systems.

## Author contributions

All authors listed have made a substantial, direct, and intellectual contribution to the work and approved it for publication.

## References

[B1] Bapat V. A. Jagtap U. B. Suprasanna P. (2022). Medicinal phytometabolites synthesis using yeast bioengineering platform. Nucleus 65, 391–397. doi: 10.1007/s13237-022-00396-1

[B2] Bapat V. A. Kavi Kishor P. B. Jalaja N. Jain S. M. Penna S. (2023). Plant cell cultures: biofactories for the production of bioactive compounds. Agronomy 13 (3), 858. doi: 10.3390/agronomy13030858

[B3] Ghag S. B. Adki V. S. Ganapathi T. R. Bapat V. A. (2021). Plant platforms for efficient heterologous protein production. Biotechnol. Bioprocess. Eng. 26, 546–567. doi: 10.1007/s12257-020-0374-1 34393545 PMC8346785

[B4] Gomes C. Silva A. C. Marques A. C. Sousa Lobo J. Amaral M. H. (2020). Biotechnology applied to cosmetics and aesthetic medicines. Cosmetics 7 (2), 33. doi: 10.3390/cosmetics7020033

[B5] Lee J. Lee S. K. Park J. S. Lee K. R. (2023). Plant-made pharmaceuticals: exploring studies for the production of recombinant protein in plants and assessing challenges ahead. Plant Biotechnol. Rep. 17 (1), 53–65. doi: 10.1007/s11816-023-00821-0 36820221 PMC9931573

[B6] Lomonossoff GP D'Aoust MA (2016). Plant-produced biopharmaceuticals: A case of technical developments driving clinical deployment. Science 353 (6305), 1237–40. 27634524 10.1126/science.aaf6638

[B7] Morikawa T. Tamura S. Wang T. (2020). Editorial: discovery and total synthesis of bio-functional natural products from traditional medicinal plants. Front. Chem. 8. doi: 10.3389/fchem.2020.00650 PMC7472932 32974270

[B8] Pham J. V. Yilma M. A. Feliz A. Majid M. T. Maffetone N. Walker J. R.. (2019). A review of the microbial production of bioactive natural products and biologics. Front. Microbiol. 10. doi: 10.3389/fmicb.2019.01404 PMC6596283 31281299

[B9] Schillberg S. Raven N. Spiegel H. Rasche S. Buntru M. (2019). Critical analysis of the commercial potential of plants for the production of recombinant proteins. Front. Plant Sci. 10. doi: 10.3389/fpls.2019.00720 PMC6579924 31244868

